# Molecular characterization of antimicrobial resistance and virulence genes of *Escherichia coli* isolates from bovine mastitis

**DOI:** 10.14202/vetworld.2020.1588-1593

**Published:** 2020-08-14

**Authors:** Zuhair Bani Ismail, Sameeh M. Abutarbush

**Affiliations:** Department of Veterinary Clinical Sciences, Faculty of Veterinary Medicine, Jordan University of Science and Technology, Irbid, Jordan

**Keywords:** antimicrobial resistance, dairy cows, environmental mastitis pathogens, *Escherichia coli*

## Abstract

**Background and Aim:**

Mastitis is a common and economically important disease in dairy cattle. It remains one of the most common reasons for the extensive use of antimicrobials in dairy farms leading to the emergence of antimicrobial-resistant pathogens. The aim of this study was to determine the patterns of antimicrobial resistance of *Escherichia coli* isolates from bovine mastitis and to identify prominent antimicrobial resistance and virulence genes among isolated strains.

**Materials and Methods:**

Antimicrobial susceptibility testing against six antibiotic groups, including tetracyclines, aminoglycosides, beta-lactams, macrolides, sulfonamides, and fluoroquinolones was performed using the disk diffusion method. PCR was performed on resistant isolates to detect resistance and virulence genes using commercially available primers.

**Results:**

Out of 216 milk samples cultured, 14 samples yielded *E. coli* isolates. All isolates (100%) were resistant to ampicillin, amoxicillin, procaine penicillin, streptomycin, oxytetracycline, and sulfamethoxazole-trimethoprim. Only one isolate (7%) was sensitive to gentamicin, and all isolates (100%) were sensitive to enrofloxacin and ciprofloxacin. All isolates carried at least one resistance gene against one or more of the major antibiotic groups. All isolates carried the *ereA, tetG, tetE*, and *tetB* genes, followed by *tetA* (93%), *ampC* (86%), *strA* (86%), *sul1* (78%), *tetD* (71%), *tetC* (57%), *aadA* (57%), and *strB* (36%). The lowest percentage of isolates carried *bla1* (17%) and bla2 (12%) genes, and none of the isolates carried the *qnrA* gene. Most of the isolates (93%) carried the Shiga toxin 1 virulence gene, followed by complement resistance protein (79%), intimin (64%), Shiga toxin 2 (36%), cytotoxic necrotizing factor (35%), aerotaxis receptor (21%), and type 1 fimbriae (15%).

**Conclusion:**

Results of this study indicate that the high percentages of *E. coli* isolate from bovine mastitis are resistant to two or more of the major antibiotic groups, irrespective of the presence or absence of relevant resistance or virulence genes.

## Introduction

Mastitis is one of the most economically important diseases of dairy cattle [1,2]. Economic losses are due to discarded milk, reduced milk production, costs of veterinary services and medications, and death or early culling of affected cows [1,2]. Mastitis is also a cause of serious concern to the well-being of cows and is considered a threat to public health [1,2]. *Escherichia coli* is considered one of the most important causes of clinical and subclinical mastitis in dairy cows [3]. Clinical signs of *E. coli* mastitis range from severe peracute disease to subclinical and chronic infection, depending on the virulence of the invading strain and immune responses of affected cows [3].

*E. coli* is a rod-shaped, flagellated, facultative anaerobic, non-sporulating, and Gram-negative bacterium of the *Enterobacteriaceae* family. Several *E. coli* isolates from raw milk and dairy products are known to cause severe foodborne illnesses in humans, including hemolytic uremic syndrome, thrombotic thrombocytopenic purpura, hemorrhagic colitis, and bloody diarrhea [4-11]. Among many virulence genes identified in *E. coli* isolated from bovine clinical mastitis, Shiga toxins (*Stx1* and *Stx2*), and *eae* (intimin) remain the most significant genes with great public health concern [5,6]. The emergence of the antimicrobial-resistant *E. coli* strains isolated from human clinical samples, and dairy cow mastitis is increasing at an alarming pace causing worldwide public health concerns [12,13]. There is strong scientific evidence that connects the development of resistance in *E. coli* strains isolated from human clinical samples and those isolated from animals [14-18].

The aim of this study was to characterize the antimicrobial resistance patterns and to identify different resistance and virulence genes in *E. coli* strain isolated from bovine acute mastitis in Jordan.

## Materials and Methods

### Ethical approval and informed consent

This study was carried out after approval by the institutional animal care, and use committee of Jordan University of Science and Technology was obtained (Grant Number 399-2019). Appropriate farm owner consents were obtained in written forms before the samples were obtained from affected cows.

### Sample collection

A total of 216 quarter milk samples were collected from 161 dairy cows with acute clinical mastitis from June to August 2019. Cows belonged to 10 farms located in the North Eastern region of Jordan. Affected cows were subjected to a complete physical examination, including heart rate, respiration rate, rectal temperature, and rumen motility (Data are not presented here). Examination of the mammary glands was carried out and included palpation of the udder and teats (to detect heat, pain, or swelling), followed by inspection of milk secretion (for color and consistency). Once the diagnosis of clinical mastitis was confirmed, approximately 100 mL of milk samples were collected from affected quarters using an aseptic technique, according to the previously published procedures [19]. The samples were transferred to the laboratory at 4°C within 3-4 h after collection.

### Bacterial culture and identification

Approximately 0.1 mL of raw milk were spread evenly on 5% sheep’s blood agar, MacConkey, and EMB agar (Merck, Germany) plates and incubated at 37°C for 24 h. Suspected *E. coli* colonies were initially identified phenotypically based on the formation of green metallic sheen on EMB. Suspected *E. coli* colonies were then confirmed biochemically using Gram’s stain, catalase, oxidase, triple sugar iron agar, and IMViC tests [7]. Further confirmation of *E. coli* was carried out using PCR to detect the *16S rRNA* gene [20].

### Antimicrobial sensitivity testing

The antimicrobial sensitivity test was performed using the Kirby-Bauer disk diffusion method on Mueller-Hinton agar (Sigma-Aldrich, USA), according to the previously published procedures [21]. The antimicrobial agents tested were amoxicillin (10 mg), ampicillin (10 mg), procaine penicillin G (10 IU), streptomycin (10 mg), gentamicin (10 mg), erythromycin (15 mg), ciprofloxacin (5 mg), oxytetracycline (30 mg), trimethoprim/sulfamethoxazole (25 mg), and enrofloxacin (5 mg). *E. coli* strain American Type Culture Collection (ATCC) 25922 was used as a reference strain. Plates were incubated at 37°C for 24 h, and the sensitivity patterns were read and reported according to the diameter of the inhibition zone as sensitive, intermediate, or resistant [21].

### DNA extraction and PCR amplification

DNA extraction was performed on all *E. coli* isolates that were phenotypically resistant on the disk diffusion sensitivity test. Bacterial DNA was extracted using Qiagen DNeasy Blood and Tissue Kit (Life Technologies, USA), according to the manufacturer’s instructions. The quality and quantity of isolated DNA were verified using NanoDrop 2000c (Thermo Fischer Scientific, USA) and stored at −20^o^C until further use.

PCR analysis was performed to detect 15 resistance and seven virulence genes using previously published primers (Tables-1 and 2) [2,22]. Each PCR reaction was performed in a volume of 25 μL that contained the following substrates: 1.5 μL MgCl_2_, 2.5 μL PCR buffer, 200 μM dNTPs, 1 μM each primer, 5 U of Taq DNA polymerase, and 1 μL (50–200 ng/μL) of test DNA. The PCR machine was set using the following program: Denaturation for 5 min at 95°C, then 30 cycles of 1 min at 94°C, 60 s at 52–58°C, and 1 min at 72°C. The final extension step was set to 10 min at 72°C. The resultant PCR products were viewed using 1.5% agarose gel using electrophoresis.

**Table-1:** Oligonucleotide primers used to detect antimicrobial resistance genes.

Antibiotic group	Target genes	Primer sequence	Size (bp)
Tetracyclines	*tetA*	F: GGCCTCAATTTCCTGACG	372
		R: AAGCAGGATGTAGCCTGTGC	
	*tetB*	F: GAGACGCAATCGAATTCGG	228
		R: TTTAGTGGCTATTCTTCCTGCC	
	*tetC*	F: TGCTCAACGGCCTCAACC	397
		R: AGCAAGACGTAGCCCAGCG	
	*tetD*	F: GGATATCTCACCGCATCTGC	436
		R: CATCCATCCGGAAGTGATAGC	
	*tetE*	F: TCCATACGCGAGATGATCTCC	442
		R: CGATTACAGCTGTCAGGTGGG	
	*tetG*	F: CAGCTTTCGGATTCTTACGG	844
		R: GATTGGTGAGGCTCGTTAGC	
Aminoglycosides	*strA*	F: CTTGGTGATAACGGCAATTC	548
		R: CCAATCGCAGATAGAAGGC	
	*strB*	F: ATCGTCAAGGGATTGAAACC	509
		R: GGATCGTAGAACATATTGGC	
	*aadA*	F: GTGGATGGCGGCCTGAAGCC	525
		R: AATGCCCAGTCGGCAGCG	
Beta-lactams	*ampC*	F: TTCTATCAAMACTGGCARCC	1048
		R: CCYTTTTATGTACCCAYGA	
	*bla1*	F: TCGCCTGTGTATTATCTCCC	768
		R: CGCAGATAAATCACCACAATG	
	*bla2*	F: TGGCCAGAACTGACAGGCAAA	462
		R: TTTCTCCTGAACGTGGCTGGC	
Macrolides	*ereA*	F: GCCGGTGCTCATGAACTTGAG	419
		R: CGACTCTATTCGATCAGAGGC	
Sulfonamides	*sul1*	F: TTCGGCATTCTGAATCTCAC	822
		R: ATGATCTAACCCTCGGTCTC	
Fluoroquinolones	*qnrA*	F: ATTTCTCACGCCAGGATTTG	516
		R: GATCGGCAAAGGTTAGGTCA	

**Table-2:** Oligonucleotide primers used to detect virulence genes.

Target genes	Target protein	Primer sequence	Size (bp)
*eaeA*	Intimin	F: ATATCCGTTTTAATGGCTATCT	425
		R: AATCTTCTGCGTACTGTGTTCA	
*aer*	Aerotaxis receptor	F: TACCGGATTGTCATATGCAGACCGT	602
		R: AATATCTTCCTCCAGTCCGGAGAAG	
*traT*	Complement resistance protein	F: GATGGCTGAACCGTGGTTATGCACA	307
		R: CGGGTCTGGTATTTATGC	
*stx1*	Shiga toxins 1	F: ATAAATCGCCATTCGTTGACTACAG	180
		R: AACGCCCACTGAGATCATCGGCACT	
*stx2*	Shiga toxins 2	F: GTCTGAAACTGCTCCTCGCCAGTTA	255
		R: TCGCCAGTTATCTGACATTCTG	
*fimH*	Type 1 fimbriae	F: TGCAGAACGGATAAGCCGTGG	508
		R:GCAGTCACCTGCCCTCCGGTA	
*Cnf*	Cytotoxic necrotizing factor	F: CTGGACTCGAGGTGGTGG	533
		R: CTCCTGTCAACCACAGCC	

## Results

Out of 216 milk samples cultured, 14 isolates were positively identified as *E. coli*. All isolates (100%) were resistant to ampicillin, amoxicillin, procaine penicillin, streptomycin, oxytetracycline, and sulfamethoxazole-trimethoprim. Only one isolate (7.14%) was sensitive to gentamicin and all isolates (100%) were sensitive to enrofloxacin and ciprofloxacin (Table-3).

**Table-3:** Numbers and percentages (%) of sensitive, intermediate, and resistant *Escherichia coli* strains isolated from bovine mastitis against common antibiotic agents (n=14).

Antibiotic agents	Sensitive	Intermediate	Resistant
Gentamicin (10 mg)	1 (7.14%)	3 (21.43)	10 (71.43)
Enrofloxacin (5 mg)	14 (100)	00	00
Ciprofloxacin (5 mg)	14 (100)	00	00
Ampicillin (10 mg)	00	00	14 (100)
Amoxicillin (10 mg)	00	00	14 (100)
Procaine penicillin (10 IU)	00	00	14 (100)
Streptomycin (10 mg)	00	00	14 (100)
Oxytetracycline (30 mg)	00	00	14 (100)
Sulfamethoxazole-Trimethoprim (25 mg)	00	00	14 (100)

**Figure-1:**
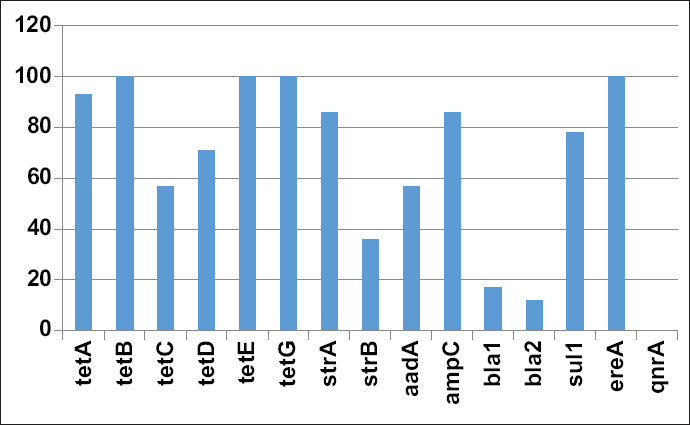
Frequency (%) of *E. coli* isolates from bovine mastitis carrying resistance genes against major antibiotic groups.

All isolates carried at least one resistance gene against one or more of the major antibiotic groups under study (Figure-1). One hundred percent of the isolates carried the *ereA, tetG, tetE*, and *tetB* genes, followed by *tetA* (93%), *ampC* (86%), *strA* (86%), *sul1* (78%), *tetD* (71%), *tetC* (57%), *aadA* (57%), and *strB* (36%). The lowest percentage of isolates carried *bla1* (17%) and *bla2* (12%) genes and none of the isolates carried the *qnrA* gene.

**Figure-2:**
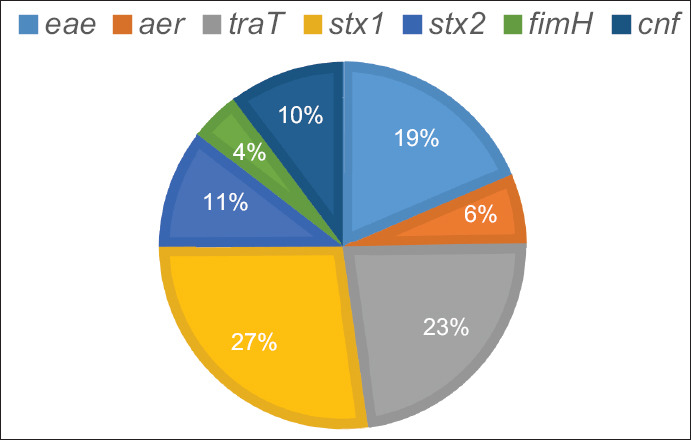
Frequency (%) of *E. coli* isolates from bovine mastitis carrying different virulence genes.

The results of the PCR detection of various virulence genes are presented in Figure-2. Most of the isolates (93%) carried the Shiga toxin 1 virulence gene, followed by complement resistance protein (79%), intimin (64%), Shiga toxin 2 (36%), cytotoxic necrotizing factor (35%), aerotaxis receptor (21%), and type 1 fimbriae (15%).

## Discussion

In this study, 100% of isolated *E. coli* strains from mastitis in dairy cows were resistant to seven out of ten different antibiotic agents belonging to six different major groups of antimicrobials. These findings are alarming and indicate a possible association between various antimicrobial resistance genes causing widespread resistance among bacterial strains isolated from dairy cows. It has been suggested that selective use of certain antibiotics in animal production has led to the emergence and dissemination of bacterial strains carrying resistance genes against multiple antibacterial agents [23]. Therefore, accurate diagnosis, judicious use of antimicrobials, and the use of a most sensitive antimicrobial agent to treat infectious diseases must be practiced in agricultural operations to limit the emergence and spread of multidrug-resistant pathogens among animals as well as humans.

The antibiotic resistance profile of *E. coli* presented in this study indicates that all isolates carried most of the genes responsible for resistance against major antimicrobial groups. Multidrug-resistant *E. coli* has been isolated from both animal and human clinical samples with an increasing rate [24]. The different levels of the resistance of *E. coli* have been reported against common antimicrobial agents, including penicillin, amoxicillin, ampicillin, streptomycin, tylosin, oxytetracycline, erythromycin, and neomycin [25]. Among various studies, differences in assay sensitivity, laboratory culture procedures and conditions, bacterial identification procedures, sampling protocols, and sample sources, and the previous exposure to different antimicrobials all may lead to variation in sensitivity patterns.

In this study, all isolates carried the *ereA, tetG, tetE*, and *tetB* genes, followed by *tetA* (93%), *ampC* (86%), *strA* (86%), *sul1* (78%), *tetD* (71%), *tetC* (57%), *aadA* (57%), and *strB* (36%). The lowest percentage of isolates carried bla1 (17%) and bla2 (12%) genes, and none of the isolates carried the *qnrA* gene. These results are similar to previously reported findings, in which various resistance gene combinations were reported in isolated *E. coli*. In one study, 78% of isolated *E. coli* strains from mastitis in cows were resistant to more than one antibiotic and carried multiple resistance genes, including *tetA, tetB, ampC tetD, tetE*, and *tetG* [2]. However, unlike our results, none of the *E. coli* in that study carried the *bla1* and only a small percentage carried the *bla2* genes [2]. In general, it has been suggested that resistant bacterial strains do not necessarily carry the genes responsible for the conferred resistance, which may suggest the presence of other mechanisms of resistance in such isolates [26].

In this study, all isolates were susceptible to both antibiotic agents belonging to fluoroquinolones. These results are in line with the previous findings, in which all *E. coli* isolates from bovine clinical mastitis were sensitive to enrofloxacin [25]. The resistance against quinolones has been described to be acquired either by mutations, downregulation, or modification of the efflux pumps activity or by the acquisition of plasmid resistance genes (*qnr* proteins) [27]. The plasmid-acquired quinolone resistance, however, has been described to lead to a low level of resistance against fluoroquinolones [28]. The fact that none of the isolates carried the *qnr* gene and all isolates were phenotypically sensitive indicates that the bacteria lack the ability to effectively develop resistance against this group of antimicrobials.

The virulence gene profile in *E. coli* isolates in this study was variable. Most of the isolates were found to carry the Shiga toxin 1, complement resistance protein, intimin, Shiga toxin 2, and cytotoxic necrotizing factor. Similar results were reported previously, in which most of the *E. coli* strains isolated from mastitic milk were found to carry the genes for complement resistance protein (*traT*), Shiga toxin 1 (*stx1*), Shiga toxin 2 (*stx2*), and intimin (*eaeA*) [2]. Other studies, however, have not detected *stx1* and *stx2* genes in any of the *E. coli* isolates [29]. It is worth to note here that a direct relationship has also been found between the presence of intimin gene and the ability of *E. coli* to cause serious disease in human beings [25]. Another intriguing finding in this study is the detection of both *stx1* and *stx2* genes in most of isolates. It is well known that these genes are present in Shiga toxin-producing *E. coli* (STEC) and are considered the primary virulence genes responsible for serious public health threats [30]. In Jordan, high rates of MDR pathogenic *E. coli* strains have been isolated from patients suffering from urinary tract infections [31]. These uropathogenic *E. coli* strains were mostly positive for *ST131* and *blaCTX-M* genes [31]. In another study, high rates of MDR pathogenic *E. coli* strains, including extended-spectrum beta-lactamase (ESBL) and carbapenemase-producing strains were recovered from different water sources in Jordan [32]. Therefore, the results of this study are in congruence with the previous findings in Jordan, indicating a continuing urgency for stricter regulations of antimicrobial use in dairy animals [33]. Indeed, the previous results obtained in one large study involving 43 dairy herds from Northwestern Jordan concluded that antimicrobials are frequently misused in dairies leading to the emergence of MDR strains of commensal *E. coli* [33].

## Conclusion

The results of this study indicate that the high percentage of *E. coli* isolates from bovine acute mastitis is resistant to two or more antibiotic groups, irrespective of the presence or absence of relevant resistance and virulence genes. These results raise a red flag on the overzealous use of antimicrobials in the dairy sector and provide a better understanding of pathogen dynamics in the field, which ultimately will improve the treatment outcome of infectious diseases.

## Authors’ Contributions

ZBI designed the study, collected samples, performed data analysis, wrote the manuscript and managed correspondence. SMA performed data analysis and interpretation, edited the manuscript. Both authors read and approved the final manuscript.

## Competing Interests

The authors declare that they have no competing interests.

## Publisher’s Note

Veterinary World remains neutral with regard to jurisdictional claims in published institutional affiliation.
